# Mr. Okes's New Styptic Powder

**Published:** 1807-09

**Authors:** Robert John Okes


### *To the Editors of the Medical and Physical Journal.*

GENTLEMEN,

I Wish to make known, by means of your Journal, a Styptic Powder against external bleeding from *small* blood-vessels. It is a good application to stop the bleeding from leeches, when necessary; it has been found useful in various bleedings from small vessels, and particularly in an alarming bleeding from ulceration of the corpora cavernosa. Its composer designed it as a solid form of the ingredients of the tinct. antiphthisica.

℞. Ferri vitriolati (sal martis.) Cerusæ acetatæ pondera æqualia, in mortario marmoreo misce, liquescentem materiam modico calore sicca, & in pulverem satis subtilem tere.

In very slight cases, a small quantity of the powder, with a bit of lint and sticking plaster, will suffice. When the bleeding is more profuse, the powder must be applied pretty thickly, (the more so the better,) lint spread with unguentum resinæ, or something of that kind, must be put over it, and secured by sticking plaster, and a proper pressure must be made. The powder forms with the effused blood a firm body, capable of resisting any farther effusion from small vessels; but it is necessary that the blood should be prevented from flowing through the powder at first by some ointment spread on lint, and pressure.

I make no doubt that many persons will suppose that lint and pressure would be equally effectual without the powder, but on trial it will be found otherwise.

There is one inconvenience attending the use of this powder, that if the wound is wiped quite clean, the skin close to the edges will be tinged of a reddish colour, which will remain a considerable time, perhaps a year; but this is easily avoided. I am, &c.

*July* 26, 1807. ROBERT JOHN OKES.

